# Two‐year trajectories of COVID‐19 symptoms and their association with illness perception: A prospective cohort study in Amsterdam, the Netherlands

**DOI:** 10.1111/irv.13190

**Published:** 2023-10-01

**Authors:** Elke Wynberg, Anouk Verveen, Hugo D. G. van Willigen, Pythia Nieuwkerk, Udi Davidovich, Anja Lok, Menno D. de Jong, Godelieve J. de Bree, Tjalling Leenstra, Hans Knoop, Maria Prins, Anders Boyd, the RECoVERED Study Group, Ivette Agard, Jane Ayal, Floor Cavdar, Marianne Craanen, Annemarieke Deuring, Annelies van Dijk, Maartje Dijkstra, Ertan Ersan, Laura del Grande, Joost Hartman, Nelleke Koedoot, Romy Lebbink, Dominique Loomans, Agata Makowska, Tom du Maine, Ilja de Man, Amy Matser, Lizenka van der Meij, Marleen van Polanen, Maria Oud, Clark Reid, Leeann Storey, Marc van Wijk, Joost van den Aardweg, Joyce van Assem, Marijne van Beek, Orlane Figaroa, Leah Frenkel, Marit van Gils, Xiaochuan ( Alvin) Han, Agnes Harskamp‐Holwerda, Mette Hazenberg, Soemeja Hidad, Nina de Jong, Neeltje Kootstra, Lara Kuijt, Eric P. Moll van Charante, Colin Russell, Karlijn van der Straten, Annelou van der Veen, Bas Verkaik, Gerben‐Rienk Visser

**Affiliations:** ^1^ Department of Infectious Diseases Public Health Service of Amsterdam Amsterdam the Netherlands; ^2^ Department of Medical Microbiology & Infection Prevention, Amsterdam UMC University of Amsterdam, Amsterdam Institute for Infection and Immunity Amsterdam the Netherlands; ^3^ Department of Medical Psychology, Amsterdam UMC, Amsterdam Public Health Research Institute University of Amsterdam Amsterdam the Netherlands; ^4^ Department of Psychiatry, Amsterdam UMC University of Amsterdam Amsterdam the Netherlands; ^5^ Department of Infectious Diseases, Amsterdam UMC, University of Amsterdam Amsterdam Institute for Infection and Immunity Amsterdam the Netherlands; ^6^ Center for Infectious Disease Control (LCI) National Institute for Public Health and the Environment (RIVM) Bilthoven Netherlands; ^7^ Stichting HIV Monitoring Amsterdam the Netherlands; ^8^ Public Health Service of Amsterdam Amsterdam the Netherlands; ^9^ Amsterdam University Medical Centres Amsterdam the Netherlands

**Keywords:** COVID‐19, illness perception, sequelae, symptoms, trajectories

## Abstract

**Background:**

We used data from a prospective cohort to explore 2‐year trajectories of ‘long COVID’ (persistent symptoms after SARS‐CoV‐2 infection) and their association with illness perception.

**Methods:**

RECoVERED participants (adults; prospectively enrolled following laboratory‐confirmed SARS‐CoV‐2 infection, May 2020–June 2021) completed symptom questionnaires at months 2–12, 18 and 24, and the Brief Illness Perception Questionnaire (B‐IPQ) at months 1, 6 and 12. Using group‐based trajectory models (GBTM), we modelled symptoms (mean total numbers and proportion with four specific complaints), including age, sex, BMI and timing of infection as covariates. In a multivariable linear mixed‐effects model, we assessed the association between symptom trajectories and repeated B‐IPQ scores.

**Results:**

Among 292 participants (42% female; median age 51 [IQR = 36–62]), four trajectories were identified, ranging from Trajectory 4 (8.9%; 6 + symptoms) to Trajectory 1 (24.8%; no symptoms). The occurrence of fatigue and myalgia increased among 23% and 12% of participants, respectively. Individuals in Trajectory 4 experienced more negative adjusted B‐IPQ scores over time than those in Trajectories 1–3.

**Conclusions:**

We observed little fluctuation in the total number of symptoms, but individual symptoms may develop as others resolve. Reporting a greater number of symptoms was congruent with more negative illness perception over time.

## 1 BACKGROUND

Millions worldwide have been infected with SARS‐CoV‐2,^1^ of whom an estimated 12%–75% develop long‐lasting symptoms,^2^, ^3^ also known as ‘long COVID’ or post‐acute sequelae of COVID‐19 (PASC). The World Health Organisation (WHO) defines long COVID as reporting one or more symptoms beyond 3 months after infection, which last for at least 2 months and cannot be explained by an alternative cause.^4^ As a consequence, most studies on long COVID focus on the duration of long COVID symptoms. However, data suggest that trajectories of long COVID symptoms may vary over time.^5^, ^6^ Some individuals may experience repeated cycles or fluctuation in long COVID symptoms, implying long COVID could present as a relapsing–remitting condition. Among these individuals, the duration of long COVID symptoms could be underestimated if the first date that symptoms are absent is defined as recovery. Furthermore, research to date has not consistently complemented data on duration of symptoms with information on the type, total number or severity of symptoms.

Standardised questionnaires that help measure subjective features of the lived experience of long COVID have also been under‐utilised. Examining illness perception builds a bridge between patient perspectives and clinical outcomes, providing detailed insight into the role of the lived experience in reporting symptom data. For other long‐term conditions, for instance, a more optimistic illness perception has been independently linked to improved prognosis, including lower mortality,^7^ whilst more negative illness perception has been associated with stress and reduced quality of life.^8^ Illness perception may be an important determining factor of the progression of long COVID symptoms over time, and therefore may help further characterise the condition and identify individuals most severely affected.

We used data from a prospective cohort of individuals infected with SARS‐CoV‐2 to identify longitudinal trajectories of long COVID symptoms over 2 years of follow‐up. We assessed baseline determinants of belonging to a given trajectory, and evaluated the association between the clinical trajectories identified and a validated measure of participants' own illness perceptions.

## 2 METHODS

### 2.1 Study design and population

RECoVERED is a prospective cohort study of adults with laboratory‐confirmed SARS‐CoV‐2 infection. Participants experienced mild to critical COVID‐19 and were enrolled in the study between May 2020 and June 2021 within 7 days after diagnosis or hospital admission. A small proportion of hospitalised participants were enrolled retrospectively to ensure representation of patients hospitalised during the first wave of COVID‐19 in the Netherlands. Further details of study design and population have been outlined previously.^9^ Briefly, eligibility criteria included laboratory confirmation of SARS‐CoV‐2 infection by reverse transcriptase polymerase chain reaction (RT‐PCR), age 16–85 years, residing in the municipal region of Amsterdam and adequate understanding of Dutch or English. Individuals residing in a nursing home and those with mental disorders deemed likely to interfere with adherence to study procedures were excluded. RECoVERED was approved by the medical ethical review board of the Amsterdam University Medical Centres (NL73759.018.20). All participants provided written informed consent.

### 2.2 Study procedures and outcome assessment

Socio‐demographic characteristics, physical measurements and medical history were recorded through standardised interviews conducted by trained staff during the first month of follow‐up. Participants reported symptom presence, severity and start/stop dates at Day 0 (D0), Day 7 (D7) and Month 1 (M1) of follow‐up for 20 different COVID‐19 symptoms.^10^ Participants then completed monthly standardised online questionnaires on the presence (in the preceding month) of these same 20 symptoms from M2–M12 and at M18 and M24 of follow‐up. Finally, the validated Brief Illness Perception Questionnaire (B‐IPQ),^11^, ^12^ which measures modalities of response to illness, was completed at M1, M6 and M12 of follow‐up.

For the current analysis, we included RECoVERED participants with at least one completed symptom questionnaire, and used data collected until 1 November 2022.

## 3 DEFINITIONS

Illness onset was defined as the earliest date upon which COVID‐19 symptoms were reported. WHO definitions for long COVID and acute COVID‐19 severity are noted in Supporting Information. BMI was defined in kg/m^2^ as: <25, underweight or normal weight; 25–29, overweight; ≥30, obese. The B‐IPQ^11^ was expressed both as a total score (continuous variable with no validated cut‐off values) and as sub‐scores for each domain: consequence/influence, timeline, personal control, treatment control, identity, concern, comprehension and emotions. Higher B‐IPQ scores denote a more negative illness perception whilst lower scores indicate a more benign illness perception. Hypothesising that illness perceptions of long COVID may have evolved over time, we defined two calendar time periods related to the timing of SARS‐CoV‐2 infection: the first COVID‐19 wave in the Netherlands (up to 1 June 2020) and all subsequent waves (1 June 2020 onwards).^13^

### 3.1 Statistical analysis

Among participants who completed at least one symptom survey, socio‐demographic, clinical and study characteristics were presented, stratifying participants by their initial COVID‐19 severity. To assess selection bias, characteristics of participants who did and did not complete at least one symptom survey were compared. Continuous variables were presented as median (IQR) and compared using the Kruskal–Wallis test, whilst categorical variables were displayed as *n* (%) and compared using Pearson's χ^2^ test.

We used group‐based trajectory modelling (GBTM) to identify long COVID trajectories over the 24‐month period since illness onset. Trajectories of the mean total number of long COVID symptoms reported (range: 0–20) were estimated using a finite‐mixture model with a censored normal distribution (thus, fully asymptomatic individuals contributed to 0 symptoms). We chose to model a priori at least three trajectories, assuming that one trajectory would represent participants without long COVID, and aimed to identify ≥2 further trajectories to differentiate participants with long COVID. The best‐fitting model (either three or four trajectories with a linear, quadratic or cubic function of time) was identified by comparing the Bayesian Information Criterion (BIC),^14^ conditional on entropy (i.e., measurement of how accurately the model classifies participants into different trajectories) of at least 0.6. Models containing a marginal probability of any one group at <5% were not considered further. We then modelled trajectories of the proportion of participants reporting fatigue, myalgia, loss of smell/taste and dyspnoea (the four most frequently‐reported long COVID symptoms in our cohort^9^) using a binary logistic distribution.

To study determinants of belonging to a given trajectory, the a posteriori probability of belonging to each group was calculated for each participant from the final GBTM based on the mean total number of symptoms. Study participants were then assigned to the trajectory group for which they had the highest probability of group membership. Participant characteristics were compared between trajectory groups using Kruskal–Wallis test for continuous variables and Pearson's χ^2^ test for categorical variables. Considering that group membership is based on a finite‐mixture distribution (i.e., there is misclassification of group membership), determinants of belonging to a group were modelled in a subsequent, multivariable GBTM. We included a priori the pre‐morbid risk factors age (years) at infection, sex and BMI category as well as timing of infection (first COVID‐19 wave vs. subsequent waves), from which the odds ratios (OR) and their 95% confidence intervals (CI) (comparing the odds of group membership between levels of the risk factors) could be estimated. Severity of initial COVID‐19 disease was not included in this model as it acts lies on the pathway between pre‐morbid risk factors and long COVID.

To assess the association between symptom trajectory group and illness perception, we calculated the median (IQR) B‐IPQ scores for each sub‐domain across trajectory groups at each time‐point and compared the scores using the Kruskal–Wallis test. In a post‐hoc analysis, the mean total B‐IPQ scores at M1, M6 and M12 were then modelled using a linear mixed‐effects model, stratified by trajectory group. Time since illness onset was included as both a fixed and random effect whilst allocated trajectory group and its interaction with time, sex and age (years) were added to the model as fixed effects. BMI category, initial COVID‐19 severity and timing of SARS‐CoV‐2 infection were included in the model if the likelihood ratio test (LRT) indicated a significantly improved fit. In an additional post‐hoc analysis, we assessed the potential of using total B‐IPQ scores at M1 as an early screening tool for later more severe long COVID. We used an ordered logit model to regress group‐based symptom trajectory group on total M1 B‐IPQ score (per 10‐point increase), with covariates selected as in the linear mixed‐effects model.

Analyses were performed using Stata (v15.1, StataCorp LLC, College Station, TX, USA). GBTM were estimated using the ‘traj’ plug‐in in Stata. A two‐sided *p*‐value <0.05 was considered statistically significant.

### 3.2 Sensitivity analyses

To understand the effect of our definition of long COVID symptoms on the identified trajectories, a sensitivity analysis was performed in which *all* symptoms (i.e., not only those arising <1 month of overall illness onset) were included in the outcome of the GBTM.

## 4 RESULTS

### 4.1 Description of the study population

Of 349 enrolled study participants by June 2021, 292 (83.7%; 86/292 [29.5%] mild, 127/292 [43.5%] moderate and 79/292 [27.1%] severe/critical COVID‐19) completed at least one symptom questionnaire and were included in the current analysis (Table 1). Participants who completed at least one symptom questionnaire were more likely to be of Dutch background (*p* = 0.003) and less likely to have been hospitalised due to COVID‐19 (*p* = 0.013) (Table S1). Most (264/349; 75.6%) study participants were recruited before 1 January 2021, prior to when the Alpha variant (B1.1.7) began to circulate in the Netherlands.^15^

**TABLE 1:** Socio‐demographic, clinical and study characteristics of participants enrolled between May 2020 and June 2021 who completed at least one symptom survey in the RECoVERED cohort in Amsterdam, the Netherlands.

		Initial COVID‐19 severity^a^			*p*‐Value
	Total	Mild	Moderate	Severe/critical	
	*N* = 292	*N* = 86	*N* = 127	*N* = 79	
Baseline socio‐demographic and medical characteristics					
Sex					0.25
Male	170 (58%)	44 (51%)	76 (60%)	50 (63%)	
Female	122 (42%)	42 (49%)	51 (40%)	29 (37%)	
Age, years	51.0 (36.0–62.0)	41.0 (29.0–54.0)	50.0 (34.0–62.0)	60.0 (51.0–66.0)	<0.001
BMI, kg/m^2^	26.1 (23.5–29.4)	24.5 (22.8–27.7)	26.2 (23.5–29.4)	27.4 (25.5–33.3)	<0.001
BMI category					<0.001
Normal weight	121 (41%)	50 (58%)	52 (41%)	19 (24%)	
Overweight	101 (35%)	23 (27%)	45 (35%)	33 (42%)	
Obese	68 (23%)	13 (15%)	30 (24%)	25 (32%)	
Missing	2 (1%)	0 (0%)	0 (0%)	2 (3%)	
Migration background					0.003
Dutch	183 (63%)	62 (72%)	77 (61%)	44 (56%)	
Non‐Dutch, OECD high‐income	31 (11%)	11 (13%)	16 (13%)	4 (5%)	
Non‐Dutch, OECD low/middle income	71 (24%)	10 (12%)	32 (25%)	29 (37%)	
Missing	7 (2%)	3 (3%)	2 (2%)	2 (3%)	
Smoking					0.13
Non‐smoker	182 (62%)	52 (60%)	74 (58%)	56 (71%)	
Smoker	19 (7%)	8 (9%)	10 (8%)	1 (1%)	
Ex‐smoker	88 (30%)	24 (28%)	43 (34%)	21 (27%)	
Missing	3 (1%)	2 (2%)	0 (0%)	1 (1%)	
Highest level of education					<0.001
None, primary or secondary education	41 (14%)	7 (8%)	22 (17%)	12 (15%)	
Vocational training	70 (24%)	9 (10%)	32 (25%)	29 (37%)	
University education	173 (59%)	67 (78%)	71 (56%)	35 (44%)	
Missing	8 (3%)	3 (3%)	2 (2%)	3 (4%)	
Number of COVID‐19 high‐risk comorbidities^b^					<0.001
0	160 (55%)	61 (71%)	75 (59%)	24 (30%)	
1	72 (25%)	18 (21%)	28 (22%)	26 (33%)	
2	36 (12%)	5 (6%)	16 (13%)	15 (19%)	
3 or more	24 (8%)	2 (2%)	8 (6%)	14 (18%)	
COVID‐19‐related clinical characteristics					
Symptom status at baseline					0.45
Symptomatic	287 (98%)	85 (99%)	127 (100%)	75 (95%)	
Asymptomatic	2 (1%)	1 (1%)	0 (0%)	1 (1%)	
Missing	3 (1%)	0 (0%)	0 (0%)	3 (4%)	
Hospital admission	141 (48%)	4 (5%)	60 (47%)	77 (97%)	<0.001
ICU admission	40 (14%)	0 (0%)	0 (0%)	40 (51%)	<0.001
Days from illness onset to COVID‐19 diagnosis	4 (2–10)	3 (1–8)	4 (2–11)	7 (2–11)	0.13
Days from illness onset to hospitalisation	9 (7–14)	43 (9–85)	10 (8–16)	9 (7–12)	0.10
Days from illness onset to ICU admission	10 (7–12)	NA	NA	10 (7–12)	
Received oxygen therapy before or during follow‐up	135 (46%)	0 (0%)	59 (46%)	76 (96%)	<0.001
Received steroid treatment					<0.001
No steroid	211 (72%)	86 (100%)	88 (69%)	37 (47%)	
Dexamethasone	55 (19%)	0 (0%)	27 (21%)	28 (35%)	
Other steroid	26 (9%)	0 (0%)	12 (9%)	14 (18%)	
Maximal HR, beats/min^c^	82 (72–94)	75 (67–81)	84 (76–94)	94 (79–107)	<0.001
Maximal RR, breaths/min^c^	20 (16–24)	16 (16–16)	20 (20–24)	24 (20–32)	<0.001
Lowest SpO_2_, %^c^	96 (91–98)	98 (97–99)	96 (93–98)	88 (80–90)	<0.001
Received COVID‐19 primary vaccination series					NA
Not vaccinated	24 (8%)	2 (2%)	13 (10%)	9 (11%)	
Vaccinated	232 (79%)	72 (84%)	102 (80%)	58 (73%)	
LTFU before vaccination	36 (12%)	12 (14%)	12 (9%)	12 (15%)	
Time from illness onset to first vaccination, days	244 (139–360)	203 (130–310)	241 (162–318)	372 (131–393)	NA
Died during follow‐up	1 (0%)	0 (0%)	1 (1%)	0 (0%)	NA
Number of reinfections					NA
0	222 (76%)	57 (66%)	92 (72%)	73 (92%)	
1	65 (22%)	27 (31%)	33 (26%)	5 (6%)	
2 or more	5 (1%)	2 (2%)	2 (2%)	1 (1%)	
Long COVID status at 12 weeks after illness onset					<0.001
No (recovered within 12 weeks)	115 (39%)	54 (63%)	43 (34%)	18 (23%)	
Yes (did not recover within 12 weeks)	177 (61%)	32 (37%)	84 (66%)	61 (77%)	
Study characteristics
Place of recruitment					<0.001
Non‐hospital	143 (49%)	75 (87%)	65 (51%)	3 (4%)	
Hospital	149 (51%)	11 (13%)	62 (49%)	76 (96%)	
Type of inclusion					<0.001
Prospective	209 (72%)	73 (85%)	99 (78%)	37 (47%)	
Retrospective	83 (28%)	13 (15%)	28 (22%)	42 (53%)	
Days from illness onset to inclusion in study	12 (6–51)	7 (4–12)	12 (6–32)	42 (14–89)	<0.001
Lost to follow‐up	109	26	40	43	NA

*Note*: Continuous variables presented as median (IQR) and compared using the Kruskal–Wallis test; categorical and binary variables presented as *n* (%) and compared using the Pearson χ ^2^ test (or Fisher exact test if *n* < 5). Time‐dependent outcomes not compared between groups due to bias resulting from differing follow‐up lengths.

Abbreviations: BMI, body mass index; COVID‐19, coronavirus disease 2019; HR, heart rate; ICU, intensive care unit; LTFU, lost to follow‐up; NA, not applicable; OECD, Organisation for Economic Co‐operation and Development; RR, respiratory rate; SpO_2_, oxygen saturation on room air.

^a^ Clinical severity groups defined as: mild as having an RR < 20/min and SpO_2_ on room air >94% at both D0 and D7; moderate disease as having a RR 20–30/min, SpO_2_ 90%–94% and/or receiving oxygen therapy at D0 or D7; severe disease as having a RR > 30/min or SpO_2_ < 90% at D0 or D7; critical disease as requiring ICU admission.

^b^ COVID‐related comorbidities are based on WHO Clinical Management Guidelines and include: cardiovascular disease (including hypertension), chronic pulmonary disease (excluding asthma), renal disease, liver disease, cancer, immunosuppression (excluding HIV, including previous organ transplantation), previous psychiatric illness and dementia.

^c^ Physical measurements at D0 and D7 study visits. Oxygen saturation measured on room air if possible or retrieved from ambulance records for hospitalised participants admitted on oxygen on day of enrollment.

### 4.2 Group‐based trajectories of long COVID symptoms and their determinants

**FIGURE 1:**
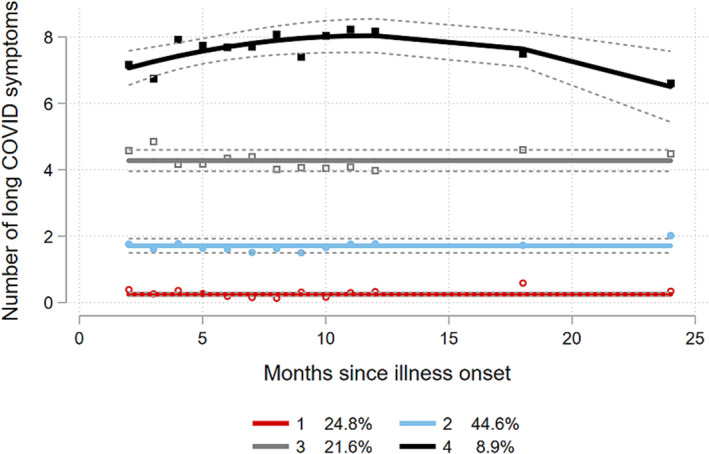
Group‐based trajectories based on mean total number of long COVID symptoms reported at 2–24 months after illness onset, adjusted for age (years), sex, BMI category and timing of infection (first wave vs. subsequent waves).

Four trajectories of long COVID symptoms were identified (Figure 1; Table S2), totalling an average of approximately 0 (i.e., no long COVID symptoms; Trajectory 1, 24.8% of study participants), 2 (Trajectory 2, 44.6%), 4 (Trajectory 3, 21.6%) or 7–8 symptoms (Trajectory 4, 8.9%) over time. Although minor fluctuation of the number of symptoms reported was observed, the overall course of symptoms was stable for each trajectory. In Trajectory 4 (highest number of symptoms, smallest proportion of participants), an increase in the mean total number of long COVID symptoms in the first year was followed by a decrease in the second year. Participants belonging to Trajectory 4 were more often female (*p* < 0.001) and less frequently had university‐level education (*p* = 0.025) compared to participants belonging to other trajectories (Table 2). A posteriori probabilities of belonging to each trajectory group are shown in Figure S2.

**TABLE 2:** Socio‐demographic and clinical characteristics of participants per long COVID symptom trajectory group.

		Symptom trajectory number				*p*‐Value
	Total	1	2	3	4	
	*N* = 290^a^	*N* = 70	*N* = 137	*N* = 59	*N* = 24	
Baseline socio‐demographic and medical characteristics						
Sex						<0.001
Male	169 (58%)	55 (79%)	75 (55%)	33 (56%)	6 (25%)	
Female	121 (42%)	15 (21%)	62 (45%)	26 (44%)	18 (75%)	
Age, years	51.0 (36.0–62.0)	46.5 (32.0–64.0)	53.0 (39.0–64.0)	51.0 (41.0–60.0)	51.0 (42.5–55.0)	0.47
BMI category						0.13
Normal weight	121 (42%)	38 (54%)	56 (41%)	20 (34%)	7 (29%)	
Overweight	101 (35%)	23 (33%)	47 (34%)	21 (36%)	10 (42%)	
Obese	68 (23%)	9 (13%)	34 (25%)	18 (31%)	7 (29%)	
Migration background						0.53
Dutch	183 (63%)	45 (64%)	87 (64%)	37 (63%)	14 (58%)	
Non‐Dutch, OECD high‐income	31 (11%)	11 (16%)	11 (8%)	7 (12%)	2 (8%)	
Non‐Dutch, OECD low/middle income	69 (24%)	12 (17%)	34 (25%)	15 (25%)	8 (33%)	
Missing	7 (2%)	2 (3%)	5 (4%)	0 (0%)	0 (0%)	
Smoking						0.82
Non‐smoker	181 (62%)	45 (64%)	83 (61%)	38 (64%)	15 (63%)	
Smoker	19 (7%)	2 (3%)	11 (8%)	5 (8%)	1 (4%)	
Ex‐smoker	87 (30%)	22 (31%)	41 (30%)	16 (27%)	8 (33%)	
Missing	3 (1%)	1 (1%)	2 (1%)	0 (0%)	0 (0%)	
Highest level of education						0.025
None, primary or secondary education	41 (14%)	8 (11%)	24 (18%)	6 (10%)	3 (13%)	
Vocational training	69 (24%)	10 (14%)	35 (26%)	13 (22%)	11 (46%)	
University education	172 (59%)	50 (71%)	72 (53%)	40 (68%)	10 (42%)	
Missing	8 (3%)	2 (3%)	6 (4%)	0 (0%)	0 (0%)	
Number of COVID‐19 high‐risk comorbidities^b^						0.73
0	160 (55%)	40 (57%)	76 (55%)	32 (54%)	12 (50%)	
1	70 (24%)	17 (24%)	30 (22%)	14 (24%)	9 (38%)	
2	36 (12%)	6 (9%)	20 (15%)	7 (12%)	3 (13%)	
3 or more	24 (8%)	7 (10%)	11 (8%)	6 (10%)	0 (0%)	
Follow‐up COVID‐19‐related characteristics						
Hospital admission	139 (48%)	24 (34%)	69 (50%)	34 (58%)	12 (50%)	0.049
ICU admission	39 (13%)	8 (11%)	20 (15%)	6 (10%)	5 (21%)	0.55
Timing of infection						0.051
First wave (up to 1 June 2020)	98 (34%)	24 (34%)	51 (37%)	21 (36%)	2 (8%)	
Subsequent waves (from 1 June 2020 onwards)	192 (66%)	46 (66%)	86 (63%)	38 (64%)	22 (92%)	
Vaccination status (primary series)						NA
Not vaccinated	24 (8%)	3 (4%)	8 (6%)	8 (14%)	5 (21%)	
Vaccinated	231 (80%)	59 (84%)	109 (80%)	47 (80%)	16 (67%)	
LTFU before vaccination	35 (12%)	8 (11%)	20 (15%)	4 (7%)	3 (13%)	
Died during follow‐up	1 (0%)	1 (1%)	0 (0%)	0 (0%)	0 (0%)	0.53
Number of reinfections						0.58
0	231 (80%)	57 (81%)	112 (82%)	45 (76%)	17 (71%)	
1	55 (19%)	13 (19%)	23 (17%)	12 (20%)	7 (29%)	
2 or more	4 (1%)	0 (0%)	2 (1%)	2 (4%)	0 (0%)	
Long COVID status at 12 weeks after illness onset						<0.001
No (recovered within 12 weeks)	121 (42%)	53 (76%)	57 (42%)	9 (15%)	2 (8%)	
Yes (did not recover within 12 weeks)	169 (58%)	17 (24%)	80 (58%)	50 (85%)	22 (92%)	

Abbreviations: BMI, body mass index; COVID‐19, coronavirus disease 2019; ICU, intensive care unit; LTFU, lost to follow‐up; NA, not applicable; OECD, Organisation for Economic Co‐operation and Development.

*Note*: Continuous variables presented as median (IQR) and compared using the Kruskal–Wallis test; categorical and binary variables presented as *n* (%) and compared using the Pearson χ ^2^ test (or Fisher exact test if *n* < 5). Trajectory group membership was based on the maximum a posteriori probability of belonging to that group. Time‐dependent outcomes not compared between groups due to bias resulting from differing follow‐up lengths.

^a^ Two individuals could not be assigned to a trajectory group due to missing risk variable (BMI).

^b^ COVID‐related comorbidities are based on WHO Clinical Management Guidelines and include: cardiovascular disease (including hypertension), chronic pulmonary disease (excluding asthma), renal disease, liver disease, cancer, immunosuppression (excluding HIV, including previous organ transplantation), previous psychiatric illness and dementia.

When modelling the finite‐mixture distribution of profile membership from the multivariable GBTM (Table 3), participants who were obese had higher odds of belonging to Trajectory 3 (*p* = 0.038) or 4 (*p* = 0.029) than Trajectory 1. Additionally, female participants had higher odds of belonging to Trajectory 2, 3 or 4 than participants in Trajectory 1 when adjusting for other covariates. Individuals infected on or after 1 June 2020 instead of during the first wave of COVID‐19 had five times higher odds of belonging to Trajectory 4 compared to Trajectory 1 (*p* = 0.046) when adjusting for other covariates, although this estimate is likely to be inflated given only two individuals infected during the first wave belonged to Trajectory 4. In a sensitivity analysis where all reported symptoms (not only those commencing <1 month of illness onset) were included, the overall course of symptom trajectories did not change, but the mean total number of symptoms reported was higher, especially for Trajectories 3 and 4 (Figure S1).

**TABLE 3:** Determinants of belonging to each trajectory based on mean total number of long COVID symptoms.

	Long COVID symptom trajectory					
	Trajectory 2 versus 1		Trajectory 3 versus 1		Trajectory 4 versus 1	
	aOR (95%CI)	*p*‐Value	aOR (95%CI)	*p*‐Value	aOR (95%CI)	*p*‐Value
Age (years)	1.01 (0.99–1.04)	0.348	1.01 (0.98–1.03)	0.635	1.01 (0.97–1.05)	0.659
BMI						
Normal weight	Ref.		Ref.		Ref.	
Overweight	1.50 (0.70–3.24)	0.298	2.08 (0.84–5.12)	0.112	4.10 (1.16–14.5)	0.029
Obese	1.65 (0.63–4.35)	0.308	3.09 (1.07–8.98)	0.038	5.03 (1.18–21.4)	0.029
Sex						
Male	Ref.		Ref.		Ref.	
Female	3.35 (1.56–7.21)	0.002	3.19 (1.34–7.56)	0.009	11.11 (3.35–36.9)	<0.001
COVID‐19 wave						
First wave	Ref.		Ref.		Ref.	
Subsequent waves	0.78 (0.37–1.65)	0.520	0.98 (0.42–2.29)	0.964	5.26 (1.03–26.9)	0.046

*Note*: BMI was defined in kg/m^2^ as: <25, underweight or normal weight; 25–29, overweight; ≥30, obese. COVID‐19 wave defined as: first wave (up to 1 June 2020) and subsequent waves (on or after 1 June 2020). Long COVID symptoms were defined as those developing within 1 month of overall illness onset, in order to exclude sporadic symptoms that were less likely to be attributed to the consequences of COVID‐19.

Abbreviations: aOR, adjusted odds ratio; CI, confidence interval.

### 4.3 Group‐based trajectories of individual symptoms

**FIGURE 2:**
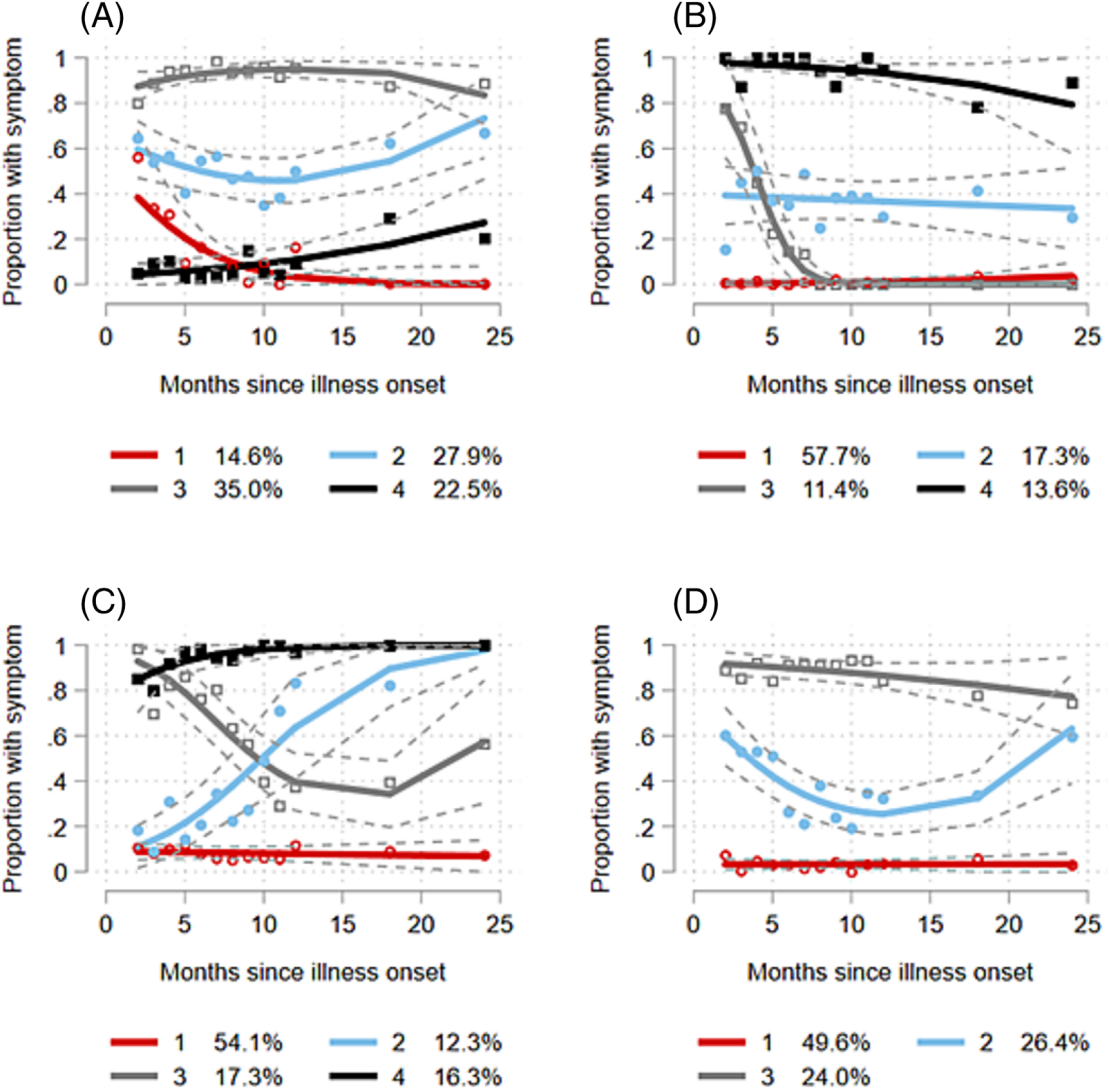
Group‐based trajectories based on the proportion reporting fatigue (A), loss of smell and/or taste (B), myalgia (C) or dyspnoea (D) at 2–24 months after illness onset, adjusted for age (years), sex, BMI category and timing of infection (first wave vs. subsequent waves).

When considering adjusted group‐based trajectories of individual symptoms, distinct patterns were observed (Figures 2A–D). Among participants with fatigue, 14.6% of participants demonstrated a steady recovery from fatigue over 2 years whilst 35.0% of participants experienced chronic fatigue after adjusting for age, sex, BMI category and timing of infection (Figure 2A). More than half of study participants (57.7%) did not report loss of smell and/or taste, and the three other trajectories of participants all demonstrated improvement over time after adjustment (either complete resolution within 10 months or a more gradual improvement; Figure 2B). Among one eighth (12.3%) of participants, the proportion reporting myalgia became gradually higher over the course of 2 years (Figure 2C). Half of study participants (49.6%) reported no dyspnoea throughout follow‐up. In the trajectory with the highest proportion of participants with dyspnoea during the first month of illness (24.0%), a steady improvement (decrease in reporting dyspnoea) was observed over the 2‐year period (Figure 2D). Tables S3–S6 show the determinants of belonging to each trajectory of fatigue, loss of smell and/or taste, myalgia and dyspnoea. When adjusting for age, sex and BMI, individuals infected on or after 1 June 2020 had significantly lower odds of experiencing moderate, chronic loss of smell/taste compared to no loss of smell/taste, than individuals infected during the first wave.

### 4.4 Association between trajectories and illness perception over time

In univariable analysis, trajectories with a higher number of long COVID symptoms had significantly higher B‐IPQ scores (i.e., more negative illness perceptions) across all sub‐domains except ‘personal control’ at M1 and except ‘treatment control’ at months 1, 6 and 12 (Table S7).

**FIGURE 3:**
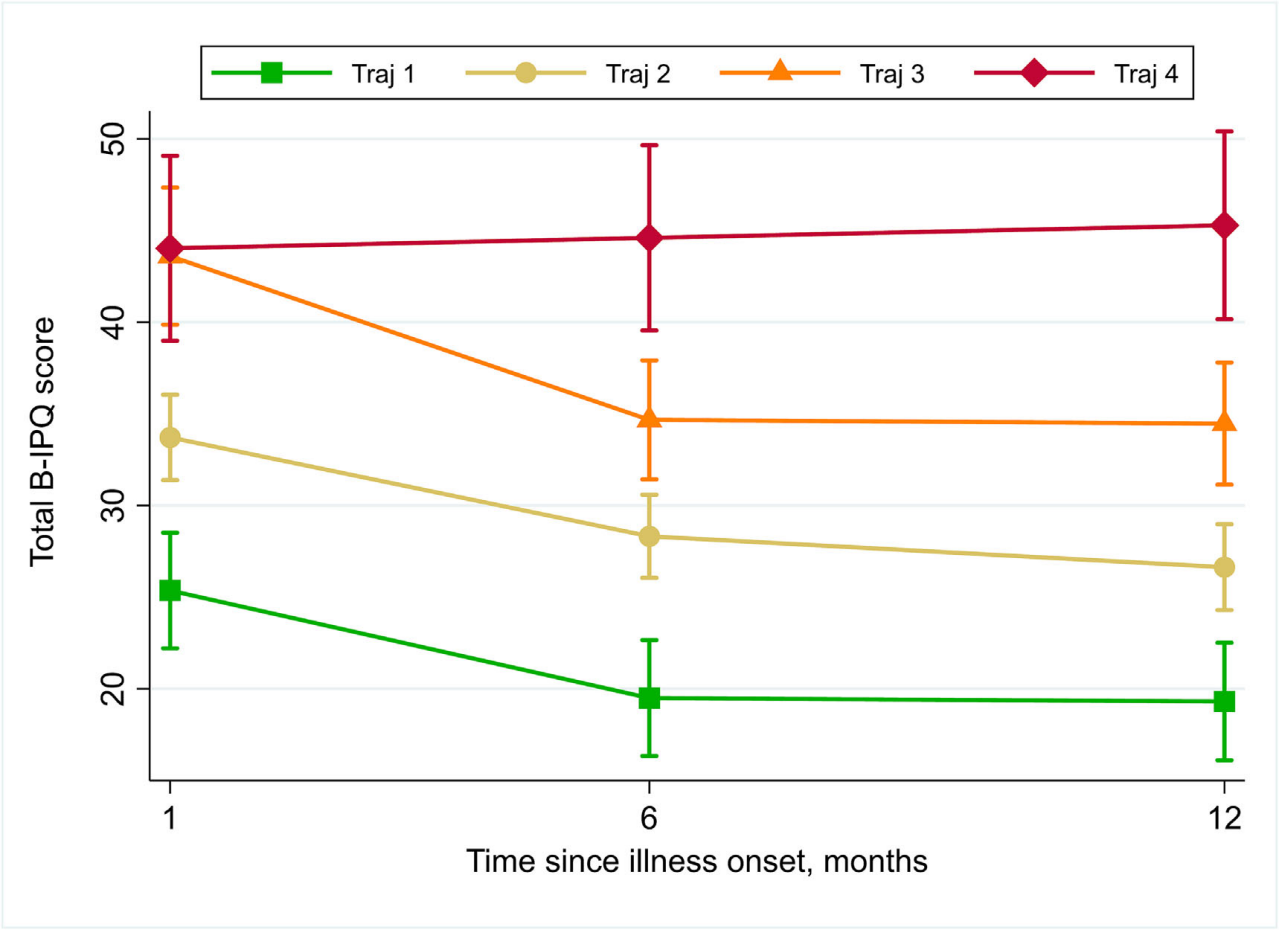
Multivariable linear mixed‐effects model of total brief illness perception (B‐IPQ) score at M1, M6 and M12 after illness onset, by 2‐year group‐based trajectory group.

In multivariable analyses, belonging to a symptom trajectory with a greater number of symptoms remained associated with higher total B‐IPQ scores over time when adjusting for age, sex, initial COVID‐19 severity and timing of infection in a linear mixed‐effects model (Figure 3; Table S8). Participants belonging to Trajectory 4 (i.e., highest number of symptoms), Trajectories 3 and 2 reported a total mean 19.2, 17.7 and 6.7 points higher B‐IPQ scores over time, respectively, than participants in Trajectory 1, when adjusting for other covariates. Participants demonstrated an average decrease (improvement) in the total B‐IPQ score over time (*p* = 0.001). Participants infected during subsequent waves had lower B‐IPQ scores compared to those infected during the first wave when adjusting for other variables (*p* < 0.001).

Finally, in a multivariable ordinal logit model, every 10‐point increase in the B‐IPQ score at M1 was associated with a 2.4 higher proportional odds (*p* < 0.001) of belonging to Trajectory 4 compared to Trajectory 1, 2 or 3 (Table S9).

## 5 DISCUSSION

In our prospective cohort based in Amsterdam, the Netherlands, we identified four distinct trajectories of long COVID symptoms over a 2‐year follow‐up since illness onset, ranging from almost no symptoms (Trajectory 1) to reporting on average 7–8 symptoms over time (Trajectory 4). Interestingly, the mean number of long COVID symptoms remained relatively stable over time in each trajectory. In contrast, trajectories of the proportion of study participants reporting individual symptoms (fatigue, loss of smell/taste, myalgia and dyspnoea) showed distinct patterns of improvement and recovery. These findings demonstrate the importance of examining the progression of specific types of symptoms alongside composite measures.

By investigating trajectories of the mean total number of long COVID symptoms over time, we aimed to shed light on both the progression of long COVID and the range of the number of symptoms that individuals with long COVID may experience. We found that approximately 1 of 10 participants in our cohort experienced more debilitating long COVID—denoted by a higher mean total number of symptoms and more negative illness perception compared to individuals with fewer symptoms. Determinants of belonging to a trajectory with a greater number of long COVID symptoms were overweight or obesity and female sex; findings highly consistent with our previous analysis on symptom recovery^9^ and research by others.^16^, ^17^, ^18^, ^19^ Our findings add value to existing evidence by demonstrating that obesity and female sex are not only risk factors for developing long COVID but also for experiencing more severe long COVID with potentially greater support and rehabilitation needs. Sex‐stratified research on long COVID may help elucidate both the biological differences and gender mechanisms underpinning sex‐based differences in long COVID risk. Although the proportion of individuals reporting 7–8 symptoms was small, the scale of the COVID‐19 pandemic (with an estimated 1 in 8 of the Dutch population experiencing long COVID^3^) means that the public health and socio‐economic consequences are likely to be substantial. Moreover, the lack of improvement observed in the total numbers of symptoms reported, even over a 24‐month period, is worrying, and suggests prognosis for overall recovery from long COVID is poor.

In contrast to the analysis of the mean total number of symptoms, the pattern of specific symptom progression was not always stable over time. These findings suggest that some individuals may experience new symptoms as other symptoms resolve. This highlights that analysis of long COVID symptoms should account for non‐linear symptom progression, to ensure that the presence of symptoms is not underestimated. Our study is not the first to explore the non‐linear progression of specific long COVID symptoms. Using Markov models, a study based in France^20^ found that the prevalence of approximately half (51%) of long COVID symptoms decreased over a 1‐year period, whereas the proportion of participants reporting parasthaesia and hair loss appeared to increase over the same period. The longer follow‐up time of our cohort allowed us to examine symptom progression beyond the first year. This revealed a relapse in the proportion of participants reporting myalgia and dyspnoea between months 12–24 after illness onset despite an overall improvement in the first year, suggesting a non‐linear course of long COVID symptoms may not be confined to the first year since illness onset.

Symptom trajectories were also highly congruent with illness perception when adjusting for age, sex, initial COVID‐19 severity and timing of infection. These findings are in line with a previous longitudinal study that found higher B‐IPQ scores at 1 year after COVID‐19 among individuals with a greater number of long COVID symptoms, reduced physical performance and higher fatigue score.^21^ Whilst we cannot proclaim a causal relationship, this association observed has two important implications. Firstly, in clinical practice, our findings imply that the B‐IPQ—a rapid assessment tool—could be used to quantify long COVID severity in real‐time, monitor individuals' progression over time or screen individuals for risk of developing more severe long COVID, as suggested by the association between the M1 B‐IPQ and belonging to a 24‐month trajectory with a greater number of symptoms. Secondly, our findings help validate using the total mean number of long COVID symptoms as a measure of subjective experience of long COVID, including differentiation of long COVID severity. Indeed, individuals belonging to Trajectory 4 scored significantly higher on all domains of the B‐IPQ except treatment control at Months 6 and 12 of follow‐up. Participants are likely to have demonstrated a negative perception of treatment control regardless of the total symptom trajectory due to the lack of treatment options for long COVID. In addition, we observed that individuals infected during the first wave tended to have more negative illness perception compared to those infected during subsequent waves, despite having lower odds of being allocated to Trajectory 4. This demonstrates that illness perception can be influenced by external factors, such as fear around COVID‐19 during the first months of the pandemic.^22^ Together, our findings infer that efforts to cultivate a more positive illness perception may help alleviate the impact of long COVID and that further investigation of utilising the B‐IPQ in clinical practice is required.

Strengths of our study include its long prospective follow‐up from illness onset onwards, representation of individuals with mild to critical COVID‐19 and detailed symptom data. Nonetheless, our study also has several limitations. First, without a SARS‐CoV‐2‐negative or pre‐pandemic historical control group, it is difficult to determine which symptoms are truly attributable to long COVID and which symptoms are due to a background prevalence of pre‐existing complaints. In an attempt to avoid counting non‐specific, transient symptoms as long COVID, we deemed symptoms arising more than 1 month after acute illness onset unrelated to long COVID. In doing so, we may have ignored some long COVID symptoms with a later date of onset. However, a sensitivity analysis removing this assumption demonstrated no difference in trajectory shapes, only in total number of symptoms. Second, our outcome of interest was captured using a list of 20 symptoms, which may not have measured the full spectrum of long COVID sequelae. As we included the most commonly‐reported long COVID symptoms, we do not expect this limitation to have had a substantial impact on the trajectories identified. Third, without validated thresholds to indicate the significance of B‐IPQ values, we cannot evaluate to what extent the differences observed in illness perception are clinically significant. Fourth, we did not include vaccination, treatment received or reinfection as time‐varying covariates in our GBTM. Reinfection may have altered the course of symptoms in a small proportion of individuals,^23^ although the impact of vaccination on long COVID symptoms appears to be negligible.^24^ As initial COVID‐19 severity determined any acute treatment received, we are unable to examine the specific effect of early treatment (during the acute phase of disease) on long COVID trajectories. In addition, efficacy data on therapeutics for long COVID are too sparse to speculate their role in these trajectories. To help inform future therapeutic trials, studies could explore the association between symptom trajectories and specific biomarkers or other outcomes that may highlight a specific pathological process.

In conclusion, we identified distinct 2‐year trajectories of long COVID symptoms, which were strongly congruent with illness perception over time. Additionally, we observed groups of individuals with diverse patterns of specific symptom progression, suggesting a need for individualised clinical management. Female sex and overweight or obesity are risk factors of having a persistently high number of symptoms and more negative B‐IPQ scores. Both researchers and clinicians should recognise, particularly when estimating the occurrence of the condition, that long COVID symptoms may fluctuate over time.

## AUTHOR CONTRIBUTIONS

Anders Boyd, Elke Wynberg and Maria Prins conceptualised the research question and analyses. Elke Wynberg and Anders Boyd developed the study methodology and conducted the formal analysis and data visualisation. Elke Wynberg, Anders Boyd, Maria Prins, Hans Knoop and Udi Davidovich drafted the initial manuscript. Elke Wynberg, Anouk Verveen and Hugo D.G. van Willigen contributed to the data collection and curation and conducted the RECoVERED cohort study project administration. Godelieve J. de Bree, Menno D. de Jong, Maria Prins and Pythia Nieuwkerk secured study financing and medical ethical approval, and conducted study supervision. All authors read a draft of the manuscript and provided feedback, contributed to interpretation of the data, and approved the final manuscript.

## CONFLICT OF INTEREST STATEMENT

The authors have no relevant conflicts of interests to declare.

### PEER REVIEW

The peer review history for this article is available at https://www.webofscience.com/api/gateway/wos/peer-review/10.1111/irv.13190.

## PATIENT CONSENT STATEMENT

Written informed consent was obtained from each study participant. The study design was approved by the local ethics committee of the Amsterdam UMC (Medisch Ethische Toetsingscommissie [METC]; NL73759.018.20).

## Supporting information


**Table S1.** Socio‐demographic, clinical and study characteristics of RECoVERED participants included and excluded from the current analyses.
**Table S2.** Bayesian Information Criteria for GBTM of total numbers of long COVID symptoms over time at 2–24 months after illness onset, according to numbers of groups and trajectory shapes.
**Table S3.** Multivariable odds ratios of belonging to each trajectory of fatigue relative to Trajectory 1, by age, BMI category, sex and timing of COVID‐19 infection.
**Table S4.** Multivariable odds ratios of belonging to each trajectory of loss of smell/taste relative to Trajectory 1, by age, BMI category, sex and timing of COVID‐19 infection.
**Table S5.** Multivariable odds ratios of belonging to each trajectory of myalgia relative to Trajectory 1, by age, BMI category, sex and timing of COVID‐19 infection.
**Table S6.** Multivariable odds ratios of belonging to each trajectory of dyspnoea relative to Trajectory 1, by age, BMI category, sex and timing of COVID‐19 infection.
**Table S7.** Median (interquartile range) illness perception sub‐domain scores by trajectory of the mean total number of long COVID symptoms.
**Table S8.** Multivariable linear mixed‐effect model of determinants of higher total illness perception questionnaire (B‐IPQ) scores over time (month 1, 6 and 12 since illness onset).
**Table S9.** Association between month 1 total B‐IPQ score and group‐based trajectory group, adjusted for age, sex and timing of SARS‐CoV‐2 infection.
**Figure S1.** Group‐based trajectories of number of any symptom reported at 2–24 months after illness onset, adjusted for age (years), sex, BMI category and timing of infection (first wave versus subsequent waves) al. Percentages in legend show the proportion of study participants belonging to the trajectory.
**Figure S2.** A posteriori probability of symptom trajectory membership, by trajectory group.

## Data Availability

Data supporting the findings in this manuscript are available from the corresponding author upon request.

## References

[1] WHO . WHO coronavirus (COVID‐19) dashboard. World Health Organization; 2022.

[2] Nasserie T , Hittle M , Goodman SN . Assessment of the Frequency and Variety of Persistent Symptoms Among Patients With COVID‐19: A Systematic Review. (2574‐3805 [Electronic]). 10.1001/jamanetworkopen.2021.11417 PMC8155823 34037731

[3] Ballering AV , van Zon SK , Olde Hartman TC , Rosmalen JG . Persistence of somatic symptoms after COVID‐19 in the Netherlands: an observational cohort study. Lancet. 2022;400(10350):452‐461. doi:10.1016/S0140-6736(22)01214-4 35934007 PMC9352274

[4] WHO . A clinical case definition of post COVID‐19 condition by a Delphi consensus. World Health Organization (WHO) clinical case definition working group on post COVID‐19 condition. 2021. 10.1016/S1473-3099(21)00703-9 PMC8691845 34951953

[5] Ladds E , Rushforth A , Wieringa S , et al. Persistent symptoms after Covid‐19: qualitative study of 114 “long Covid” patients and draft quality principles for services. BMC Health Serv Res. 2020;20(1):1144. doi:10.1186/s12913-020-06001-y 33342437 PMC7750006

[6] Ireson J , Taylor A , Richardson E , Greenfield B , Jones G . Exploring invisibility and epistemic injustice in long Covid—a citizen science qualitative analysis of patient stories from an online Covid community. Health Expect. 2022;25(4):1753‐1765. doi:10.1111/hex.13518 35557480 PMC9327841

[7] Chilcot J , Wellsted D , Fau‐Farrington K , Farrington K . Illness perceptions predict survival in haemodialysis patients. (1421‐9670 [Electronic]). 10.1159/000326752 21430374

[8] Zhang M , Hong L , Zhang T , et al. Illness perceptions and stress: mediators between disease severity and psychological well‐being and quality of life among patients with Crohn's disease. (1177‐889X [Print]). 10.2147/PPA.S118413 PMC5125764 27920505

[9] Wynberg E , van Willigen HDG , Dijkstra M , et al. Evolution of coronavirus disease 2019 (COVID‐19) symptoms during the first 12 months after illness onset. Clin Infect Dis. 2022;75(1):e482‐e490. doi:10.1093/cid/ciab759 34473245 PMC8522402

[10] ISARIC . Clinical Data Collection – The COVID‐19 Case Report Forms (CRFs). Accessed 18 February. Available at: https://isaric.org/research/covid-19-clinical-research-resources/covid-19-crf/

[11] Basu S , Fau‐Poole J , Poole J . The Brief Illness Perception Questionnaire. (1471‐8405 [Electronic]).

[12] Weinman J . Brief Illness Perception Questionnaire/Illness Perception Questionnaire‐Kort/Ziekteperceptievragenlijst. Accessed 29 November. Available at: https://meetinstrumentenzorg.nl/instrumenten/brief-illness-perception-questionnaire-illness-perception-questionnaire-kort-ziekteperceptievragenlijst/

[13] Coyer L , Wynberg E , Buster M , et al. Hospitalisation rates differed by city district and ethnicity during the first wave of COVID‐19 in Amsterdam, the Netherlands. medRxiv 2021: 2021.03.15.21253597. 10.1186/s12889-021-11782-w PMC8456400 34551752

[14] Walsh CA‐O , Mucherino S , Orlando V , Bennett KE , Menditto E , Cahir C . Mapping the use of Group‐Based Trajectory Modelling in medication adherence research: A scoping review protocol. (2515‐4826 [Electronic]). 10.12688/hrbopenres.13056.1 PMC7265572 32551416

[15] RIVM . Varianten van het coronavirus SARS‐CoV‐2. Accessed 1 April 2023. Available at: https://www.rivm.nl/coronavirus-covid-19/virus/varianten

[16] Evans RA , Leavy OC , Richardson M , et al. Clinical characteristics with inflammation profiling of long COVID and association with 1‐year recovery following hospitalisation in the UK: a prospective observational study. Lancet Respir Med. 2022;10(8):761‐775. doi:10.1016/S2213-2600(22)00127-8 35472304 PMC9034855

[17] Fernández‐de‐Las‐Peñas C , Torres‐Macho J , Velasco‐Arribas M , et al. Preexisting hypertension is associated with a greater number of long‐term post‐COVID symptoms and poor sleep quality: a case–control study. J Hum Hypertens. 2022;36(6):1‐3. doi:10.1038/s41371-022-00660-6 35173268 PMC8853057

[18] Maglietta G , Diodati F , Puntoni M , et al. Prognostic factors for post‐COVID‐19 syndrome: a systematic review and meta‐analysis. J Clin Med. 2022;11(6):1541. doi:10.3390/jcm11061541 35329867 PMC8948827

[19] Fernández‐de‐Las‐Peñas C , Martín‐Guerrero JD , Pellicer‐Valero ÓJ , et al. Female sex is a risk factor associated with long‐term post‐COVID related‐symptoms but not with COVID‐19 symptoms: the LONG‐COVID‐EXP‐CM multicenter study. J Clin Med. 2022;11(2):413. doi:10.3390/jcm11020413 35054108 PMC8778106

[20] Tran V‐T , Porcher R , Pane I , Ravaud P . Course of post COVID‐19 disease symptoms over time in the ComPaRe long COVID prospective e‐cohort. Nat Commun. 2022;13(1):1812. doi:10.1038/s41467-022-29513-z 35383197 PMC8983754

[21] Hüfner K , Tymoszuk P , Sahanic S , et al. Persistent somatic symptoms are key to individual illness perception at one year after COVID‐19 in a cross‐sectional analysis of a prospective cohort study. (1879‐1360 [Electronic]). 10.1016/j.jpsychores.2023.111234 PMC10022460 36965396

[22] Duintjer Tebbens MBS . Zo kom je zonder angst en stress de tweede golf door. NOS 2020 18 October 2020.

[23] de Siméon B , van Albert Jan H , Elizabeth NM , et al. Lower prevalence of Post‐Covid‐19 Condition following Omicron SARS‐CoV‐2 infection. medRxiv 2023: 2023.04.05.23288157.

[24] Wynberg E , Han AX , Boyd A , et al. The effect of SARS‐CoV‐2 vaccination on post‐acute sequelae of COVID‐19 (PASC): A prospective cohort study. (1873‐2518 [Electronic]). 10.1016/j.vaccine.2022.05.090 PMC9170535 35725782

