# Supplementary material for: Two‐year trajectories of COVID‐19 symptoms and their association with illness perception: A prospective cohort study in Amsterdam, the Netherlands
Source: Influenza Other Respir Viruses. 2023 Oct 1;17(10):e13190. doi: 10.1111/irv.13190 (PMC10542619; doi:10.1111/irv.13190)
Supplement: Supplementary file 1 — Table S1. Socio‐demographic, clinical and study characteristics of RECoVERED participants included and excluded from the current analyses. Table S2. Bayesian Information Criteria for GBTM of total numbers of long COVID symptoms over time at 2–24 months after illness onset, according to numbers of groups and trajectory shapes. Table S3. Multivariable odds ratios of belonging to each trajectory of fatigue relative to Trajectory 1, by age, BMI category, sex and timing of COVID‐19 infection. Table S4. Multivariable odds ratios of belonging to each trajectory of loss of smell/taste relative to Trajectory 1, by age, BMI category, sex and timing of COVID‐19 infection. Table S5. Multivariable odds ratios of belonging to each trajectory of myalgia relative to Trajectory 1, by age, BMI category, sex and timing of COVID‐19 infection. Table S6. Multivariable odds ratios of belonging to each trajectory of dyspnoea relative to Trajectory 1, by age, BMI category, sex and timing of COVID‐19 infection. Table S7. Median (interquartile range) illness perception sub‐domain scores by trajectory of the mean total number of long COVID symptoms. Table S8. Multivariable linear mixed‐effect model of determinants of higher total illness perception questionnaire (B‐IPQ) scores over time (month 1, 6 and 12 since illness onset). Table S9. Association between month 1 total B‐IPQ score and group‐based trajectory group, adjusted for age, sex and timing of SARS‐CoV‐2 infection. Figure S1. Group‐based trajectories of number of any symptom reported at 2–24 months after illness onset, adjusted for age (years), sex, BMI category and timing of infection (first wave versus subsequent waves) al. Percentages in legend show the proportion of study participants belonging to the trajectory. Figure S2. A posteriori probability of symptom trajectory membership, by trajectory group. [file IRV-17-e13190-s001.docx]

# Supplementary Files

## Methods

**Supplementary Methods** World Health Organization definitions for long COVID and acute COVID-19 severity

## Tables

**Supplementary Table S1.** Socio-demographic, clinical and study characteristics of RECoVERED participants included and excluded from the current analyses

**Supplementary Table S2.** Bayesian Information Criteria for GBTM of total numbers of long COVID symptoms over time at 2-24 months after illness onset, according to numbers of groups and trajectory shapes

**Supplementary Table S3.** Multivariable odds ratios of belonging to each trajectory of fatigue relative to Trajectory 1, by age, BMI category, sex and timing of COVID-19 infection

**Supplementary Table S4.** Multivariable odds ratios of belonging to each trajectory of loss of smell/taste relative to Trajectory 1, by age, BMI category, sex and timing of COVID-19 infection

**Supplementary Table S5.** Multivariable odds ratios of belonging to each trajectory of myalgia relative to Trajectory 1, by age, BMI category, sex and timing of COVID-19 infection

**Supplementary Table S6.** Multivariable odds ratios of belonging to each trajectory of dyspnoea relative to Trajectory 1, by age, BMI category, sex and timing of COVID-19 infection

**Supplementary Table S7.** Median (interquartile range) illness perception sub-domain scores by trajectory of the mean total number of long COVID symptoms

**Supplementary Table S8.** Multivariable linear mixed-effect model of determinants of higher total illness perception questionnaire (B-IPQ) scores over time (month 1, 6 and 12 since illness onset)

**Supplementary Table S9.** Association between month 1 total B-IPQ score and group-based trajectory group, adjusted for age, sex and timing of SARS-CoV-2 infection

## Figures

**Supplementary Figure S1.** Group-based trajectories of number of any symptom reported at 2-24 months after illness onset, adjusted for age (years), sex, BMI category and timing of infection (first wave versus subsequent waves)

**Supplementary Figure S2.** *A posteriori* probability of symptom trajectory membership, by trajectory group

**Supplementary Methods. World Health Organization definitions for long COVID and acute COVID-19 severity**

The WHO long COVID definition was used, namely: reporting ≥1 COVID-19 symptom >3 months after illness onset, and lasting for ≥2 months (i.e., started within one month of overall illness onset)[1]. In an attempt to exclude symptoms secondary to alternative explanations, we considered symptoms commencing >1 month after the onset of COVID-19 unlikely to be due to SARS-CoV-2 infection. COVID-19 severity was categorised according to WHO disease severity criteria[2]: mild disease as having a RR <20/min and SpO2>94% on room air at D0 and D7 study visits; moderate disease as having a RR 20-30/min and/or SpO2 90-94% or receiving oxygen therapy at D0 or D7; severe disease as having a RR>30/min and/or SpO2<90% or receiving oxygen therapy at D0 or D7; critical disease as ICU admission due to COVID-19 at any point.

**Supplementary Table S1. Socio-demographic, clinical and study characteristics of RECoVERED participants included and excluded from the current analyses**

|  |  | **Inclusion status in current analyses** | |  |
| --- | --- | --- | --- | --- |
|  | **Total** | **Excluded** | **Included** | **p-value** |
|  | **N=349** | **N=57** | **N=292** |  |
| **Socio-demographic and clinical characteristics at baseline** | | | | |
| Sex |  |  |  | 0.079 |
| Male | 196 (56%) | 26 (46%) | 170 (58%) |  |
| Female | 153 (44%) | 31 (54%) | 122 (42%) |  |
| Age, years | 51.0 (36.0-62.0) | 50.0 (31.0-62.0) | 51.0 (36.0-62.0) | 0.76 |
| BMI, kg/m^2^ | 26.2 (23.4-29.7) | 27.3 (23.3-30.6) | 26.1 (23.5-29.4) | 0.55 |
| BMI category |  |  |  | 0.42 |
| Normal weight | 141 (40%) | 20 (35%) | 121 (41%) |  |
| Overweight | 114 (33%) | 13 (23%) | 101 (35%) |  |
| Obese | 83 (24%) | 15 (26%) | 68 (23%) |  |
| Missing | 11 (3%) | 9 (16%) | 2 (1%) |  |
| Migration background |  |  |  | 0.003 |
| Dutch | 194 (56%) | 11 (19%) | 183 (63%) |  |
| Non-Dutch, OECD high-income | 40 (11%) | 9 (16%) | 31 (11%) |  |
| Non-Dutch, OECD low/middle income | 80 (23%) | 9 (16%) | 71 (24%) |  |
| Missing | 35 (10%) | 28 (49%) | 7 (2%) |  |
| Smoking |  |  |  | 0.91 |
| Non-smoker | 207 (59%) | 25 (44%) | 182 (62%) |  |
| Smoker | 22 (6%) | 3 (5%) | 19 (7%) |  |
| Ex-smoker | 102 (29%) | 14 (25%) | 88 (30%) |  |
| Missing | 18 (5%) | 15 (26%) | 3 (1%) |  |
| Highest level of education |  |  |  | 0.81 |
| None, primary or secondary education | 45 (13%) | 4 (7%) | 41 (14%) |  |
| Vocational training | 77 (22%) | 7 (12%) | 70 (24%) |  |
| University education | 186 (53%) | 13 (23%) | 173 (59%) |  |
| Missing | 41 (12%) | 33 (58%) | 8 (3%) |  |
| Number of COVID-19 high-risk comorbidities |  |  |  | 0.12 |
| 0 | 189 (54%) | 29 (51%) | 160 (55%) |  |
| 1 | 81 (23%) | 9 (16%) | 72 (25%) |  |
| 2 | 49 (14%) | 13 (23%) | 36 (12%) |  |
| 3 or more | 30 (9%) | 6 (11%) | 24 (8%) |  |
| **Clinical COVID-19-related characteristics during follow-up** | | | | |
| Clinical severity score |  |  |  | 0.40 |
| Mild | 99 (28%) | 13 (23%) | 86 (29%) |  |
| Moderate | 151 (43%) | 24 (42%) | 127 (43%) |  |
| Severe/critical | 99 (28%) | 20 (35%) | 79 (27%) |  |
| Hospital admission | 179 (51%) | 38 (67%) | 141 (48%) | 0.013 |
| ICU admission | 45 (13%) | 5 (9%) | 40 (14%) | 0.39 |
| Days from illness onset to COVID-19 diagnosis | 4 (2-10) | 6 (2-9) | 4 (2-10) | 0.38 |
| Days from illness onset to hospitalisation | 9 (7-14) | 8 (3-14) | 9 (7-14) | 0.23 |
| Days from illness onset to ICU admission | 10 (7-12) | 8 (6-9) | 10 (7-12) | 0.11 |
| Received oxygen therapy before or during follow-up | 169 (49%) | 34 (63%) | 135 (46%) | 0.024 |
| Maximal HR, beats/min | 83 (72-94) | 84 (72-96) | 82 (72-94) | 0.66 |
| Maximal RR, breaths/min | 20 (16-24) | 20 (16-25) | 20 (16-24) | 0.34 |
| Lowest SpO2, % | 96 (91-98) | 95 (88-98) | 96 (91-98) | 0.38 |
| Vaccinated during follow-up (primary series) |  |  |  | NA |
| Not vaccinated | 25 (7%) | 1 (2%) | 24 (8%) |  |
| Vaccinated | 233 (67%) | 1 (2%) | 232 (79%) |  |
| LTFU before vaccination | 91 (26%) | 55 (96%) | 36 (12%) |  |
| Died during follow-up | 5 (1%) | 4 (7%) | 1 (0%) | NA |
| Number of reinfections |  |  |  | NA |
| 0 | 278 (80%) | 56 (98%) | 222 (76%) |  |
| 1 | 66 (19%) | 1 (2%) | 65 (22%) |  |
| 2 or more | 5 (1%) | 0 (0%) | 5 (1%) |  |
| PASC at 12 weeks after illness onset |  |  |  | <0.001 |
| Recovered within 12 weeks | 126 (36%) | 11 (19%) | 115 (39%) |  |
| Did not recover within 12 weeks | 205 (59%) | 28 (49%) | 177 (61%) |  |
| LTFU within 12 weeks | 18 (5%) | 18 (32%) | 0 (0%) |  |
| **Study characteristics** | | | | |
| Place of recruitment |  |  |  | 0.016 |
| Non-hospital | 161 (46%) | 18 (32%) | 143 (49%) |  |
| Hospital | 188 (54%) | 39 (68%) | 149 (51%) |  |
| Type of inclusion |  |  |  | 0.048 |
| Prospective | 257 (74%) | 48 (84%) | 209 (72%) |  |
| Retrospective | 92 (26%) | 9 (16%) | 83 (28%) |  |
| Days from illness onset to inclusion in study | 12 (6-38) | 11 (7-20) | 12 (6-51) | 0.41 |
| Lost to follow-up | 164 | 55 | 109 | NA |

Abbreviations: BMI, body mass index; COVID-19, coronavirus disease 2019; HR, heart rate; ICU, intensive care unit; LTFU, lost to follow-up; OECD, Organisation for Economic Co-operation and Development; NA, not applicable; PCR, polymerase chain reaction; SpO2, oxygen saturation on room air; RR, respiratory rate; SARS-CoV-2, severe acute respiratory syndrome coronavirus 2.

Continuous variables presented as median (IQR) and compared using the Kruskal-Wallis test; categorical and binary variables presented as n(%) and compared using the Pearson χ^2^ test (or Fisher exact test if n <5).

Clinical severity groups defined as: mild as having an RR <20/min and SpO2 on room air >94% at both D0 and D7; moderate disease as having a RR 20–30/minutes, SpO2 90–94% and/or receiving oxygen therapy at D0 or D7; severe disease as having a RR >30/minutes or SpO2 <90% at D0 or D7; critical disease as requiring ICU admission.

COVID-related comorbidities are based on WHO Clinical Management Guidelines and include: cardiovascular disease (including hypertension), chronic pulmonary disease (excluding asthma), renal disease, liver disease, cancer, immunosuppression (excluding HIV, including previous organ transplantation), previous psychiatric illness and dementia.

Physical measurements at D0 and D7 study visits. Oxygen saturation measured on room air if possible or retrieved from ambulance records for hospitalized participants admitted on oxygen on day of enrollment.

Time-dependent outcomes not compared between groups (NA) due to bias resulting from differing follow-up lengths.

**Supplementary Table S2. Bayesian Information Criteria and entropy values for select unadjusted GBTM of total numbers of long COVID symptoms over time, according to numbers of groups and trajectory shapes**

| **Number of groups** | **Trajectory shapes** | **BIC (N=275)** | **% of participants in smallest trajectory group** | **Entropy** |
| --- | --- | --- | --- | --- |
| 3 | 1 1 1 | -4412 | 16.1% | 0.690 |
| 3 | 2 2 2 | -4418 | 16.1% | 0.690 |
| 3 | 3 3 3 | -4420 | 16.1% | 0.692 |
| 3 | 1 1 0 | -4410 | 16.1% | 0.690 |
| 3 | 1 1 2 | -4415 | 16.1% | 0.690 |
| 3 | 1 1 3 | -4417 | 16.1% | 0.689 |
| 4 | 0 0 0 1 | -4306 | 8.3% | 0.652 |
| 4 | 0 0 0 2 † | -4303 | 8.2% | 0.651 |
| 4 | 0 0 0 3 | -4306 | 8.2% | 0.651 |
| 4 | 0 0 1 1 | -4309 | 8.2% | 0.653 |
| 4 | 0 0 2 2 | -4306 | 8.2% | 0.654 |
| 4 | 0 0 3 3 | -4310 | 7.8% | 0.656 |

GBTM = group-based trajectory modelling. Trajectory shapes classified as: 0 (intercept-only), 1 (linear), 2 (quadratic), 3 (cubic). BIC denotes the Bayesian Information Criterion which denotes the posterior probability of a model given the data. Entropy measures how accurately the model classifies participants into different trajectories.

Limited selection of all possible combinations shown.

† Chosen model due to least negative BIC

**Supplementary Table S3. Determinants of belonging to each trajectory of fatigue relative to Trajectory 1, by age, BMI category, sex and timing of COVID-19 infection**

|  | **Trajectory of fatigue** | | | | | |
| --- | --- | --- | --- | --- | --- | --- |
|  | **“Moderate, U-shaped” vs. “Mild, recovering”** | | **“Severe, chronic” vs. “Mild, recovering”** | | **“Mild, progressive” vs. “Mild, recovering”** | |
|  | aOR (95%CI) | p-value | aOR (95%CI) | p-value | aOR (95%CI) | p-value |
| **Age (years)** | 0.90 (0.82-0.90) | 0.017 | 0.92 (0.86-1.00) | 0.045 | 0.88 (0.79-0.98) | 0.021 |
| **BMI** |  |  |  |  |  |  |
| Normal weight | Ref. | Ref. | Ref. | Ref. | Ref. | Ref. |
| Overweight | 0.10 (0.02-0.69) | 0.019 | 0.42 (0.08-2.27) | 0.312 | 0.13 (0.02-0.99) | 0.049 |
| Obese | 0.49 (0.07-3.54) | 0.481 | 0.57 (0.08-3.91) | 0.564 | 0.17 (0.01-2.89) | 0.222 |
| **Sex** |  |  |  |  |  |  |
| Male | Ref. | Ref. | Ref. | Ref. | Ref. | Ref. |
| Female | 3.65 (0.75-17.69) | 0.108 | 4.22 (1.05-16.89) | 0.042 | 1.32 (0.23-7.51) | 0.757 |
| **COVID-19 wave** |  |  |  |  |  |  |
| First wave | Ref. | Ref. | Ref. | Ref. | Ref. | Ref. |
| Subsequent waves | 1.90 (0.43-8.34) | 0.394 | 1.70 (0.49-5.88) | 0.402 | 4.25 (0.63-28.55) | 0.136 |

BMI was defined in kg/m2 as: <25, underweight or normal weight; 25-29, overweight; ≥30, obese. COVID-19 wave defined as: first wave (up to 1 June 2020) and subsequent waves (on or after 1 June 2020). Long COVID symptoms were defined as those developing within 1 month of overall illness onset, in order to exclude sporadic symptoms that were less likely to be attributed to the consequences of COVID-19.

CI= confidence interval; aOR= adjusted odds ratio.

**Supplementary Table S4. Determinants of belonging to each trajectory of loss of smell/taste relative to Trajectory 1, by age, BMI category, sex and timing of COVID-19 infection**

|  | **Trajectory of loss of smell and/or taste** | | | | | |
| --- | --- | --- | --- | --- | --- | --- |
|  | **“Moderate, chronic” vs. no loss of smell/taste** | | **“Rapid recovery” vs. no loss of smell/taste** | | **“Severe, recovering” vs. no loss of smell/taste** | |
|  | aOR (95%CI) | p-value | aOR (95%CI) | p-value | aOR (95%CI) | p-value |
| **Age (years)** | 1.02 (0.99-1.06) | 0.190 | 1.02 (0.98-1.06) | 0.446 | 1.01 (0.97-1.04) | 0.747 |
| **BMI** |  |  |  |  |  |  |
| Normal weight | Ref. | Ref. | Ref. | Ref. | Ref. | Ref. |
| Overweight | 0.97 (0.28-3.35) | 0.966 | 2.39 (0.76-7.49) | 0.136 | 1.05 (0.34-3.19) | 0.937 |
| Obese | 1.66 (0.46-6.02) | 0.438 | NA | NA | 1.75 (0.56-5.41) | 0.334 |
| **Sex** |  |  |  |  |  |  |
| Male | Ref. | Ref. | Ref. | Ref. | Ref. | Ref. |
| Female | 0.97 (0.33-2.83) | 0.955 | 0.99 (0.31-3.13) | 0.983 | 1.71 (0.67-4.41) | 0.265 |
| **COVID-19 wave** |  |  |  |  |  |  |
| First wave | Ref. | Ref. | Ref. | Ref. | Ref. | Ref. |
| Subsequent waves | 0.20 (0.07-0.58) | 0.003 | 1.21 (0.28-5.30) | 0.799 | 0.51 (0.19-1.38) | 0.187 |

BMI was defined in kg/m2 as: <25, underweight or normal weight; 25-29, overweight; ≥30, obese. COVID-19 wave defined as: first wave (up to 1 June 2020) and subsequent waves (on or after 1 June 2020). Long COVID symptoms were defined as those developing within 1 month of overall illness onset, in order to exclude sporadic symptoms that were less likely to be attributed to the consequences of COVID-19.

CI= confidence interval; aOR= adjusted odds ratio.

**Supplementary Table S5. Determinants of belonging to each trajectory of myalgia relative to Trajectory 1, by age, BMI category, sex and timing of COVID-19 infection**

|  | **Trajectory of myalgia** | | | | | |
| --- | --- | --- | --- | --- | --- | --- |
|  | **“Severe, progressive” vs. “Minimal, chronic”** | | **“Moderate, U-shaped” vs. “Minimal, chronic”** | | **“Severe, progressive” vs. “Minimal, chronic”** | |
|  | aOR (95%CI) | p-value | aOR (95%CI) | p-value | aOR (95%CI) | p-value |
| **Age (years)** | 1.02 (0.97-1.07) | 1.02 | 1.03 (1.00-1.07) | 0.058 | 1.04 (1.00-1.08) | 0.034 |
| **BMI** |  |  |  |  |  |  |
| Normal weight | Ref. | Ref. | Ref. | Ref. | Ref. | Ref. |
| Overweight | 3.22 (0.60-17.29) | 3.22 | 0.31 (0.07-1.33) | 0.114 | 1.38 (0.43-4.44) | 0.586 |
| Obese | 3.44 (0.46-25.90) | 3.44 | NA | NA | 2.05 (0.56-7.47) | 0.275 |
| **Sex** |  |  |  |  |  |  |
| Male | Ref. | Ref. | Ref. | Ref. | Ref. | Ref. |
| Female | 0.70 (0.11-4.47) | 0.70 | 3.43 (1.13-10.35) | 0.029 | 2.23 (0.79-6.31) | 0.132 |
| **COVID-19 wave** |  |  |  |  |  |  |
| First wave | Ref. | Ref. | Ref. | Ref. | Ref. | Ref. |
| Subsequent waves | 0.62 (0.14-2.63) | 0.62 | 0.39 (0.12-1.24) | 0.109 | 0.56 (0.18-1.68) | 0.300 |

BMI was defined in kg/m2 as: <25, underweight or normal weight; 25-29, overweight; ≥30, obese. COVID-19 wave defined as: first wave (up to 1 June 2020) and subsequent waves (on or after 1 June 2020). Long COVID symptoms were defined as those developing within 1 month of overall illness onset, in order to exclude sporadic symptoms that were less likely to be attributed to the consequences of COVID-19.

CI= confidence interval; aOR= adjusted odds ratio.

**Supplementary Table S6. Determinants of belonging to each trajectory of dyspnoea relative to Trajectory 1, by age, BMI category, sex and timing of COVID-19 infection**

|  | **Trajectory of dyspnoea** | | | |
| --- | --- | --- | --- | --- |
|  | **“Moderate, U-shaped” vs. no dyspnoea** | | **“Severe, recovering” vs. no dyspnoea** | |
|  | aOR (95%CI) | p-value | aOR (95%CI) | p-value |
| **Age (years)** | 1.06 (1.02-1.10) | 0.002 | 1.04 (1.01-1.08) | 0.023 |
| **BMI** |  |  |  |  |
| Normal weight | Ref. | Ref. | Ref. | Ref. |
| Overweight | 1.96 (0.65-5.86) | 0.231 | 6.58 (1.59-27.23) | 0.009 |
| Obese | 2.85 (0.90-9.04) | 0.075 | 9.38 (2.23-39.38) | 0.002 |
| **Sex** |  |  |  |  |
| Male | Ref. | Ref. | Ref. | Ref. |
| Female | 1.22 (0.44-3.40) | 0.698 | 5.00 (1.74-14.34) | 0.003 |
| **COVID-19 wave** |  |  |  |  |
| First wave | Ref. | Ref. | Ref. | Ref. |
| Subsequent waves | 2.00 (0.66-6.02) | 0.220 | 0.46 (0.16-1.28) | 0.137 |

BMI was defined in kg/m2 as: <25, underweight or normal weight; 25-29, overweight; ≥30, obese. COVID-19 wave defined as: first wave (up to 1 June 2020) and subsequent waves (on or after 1 June 2020). Long COVID symptoms were defined as those developing within 1 month of overall illness onset, in order to exclude sporadic symptoms that were less likely to be attributed to the consequences of COVID-19.

CI= confidence interval; aOR= adjusted odds ratio.

**Supplementary Table S7. Median (interquartile range) illness perception sub-domain scores by trajectory of the mean total number of long COVID symptoms**

|  |  |  |  |  |  |  |
| --- | --- | --- | --- | --- | --- | --- |
|  | **Total** | **Trajectory 1** | **Trajectory 2** | **Trajectory 3** | **Trajectory 4** | **p-value** |
| **Month 1** |  |  |  |  |  |  |
| **N** | **209** | **54** | **97** | **36** | **29** |  |
| Consequences | 5 (2-8) | 3 (1-5) | 5 (2-7) | 8 (6-9) | 8 (7-10) | <0.001 |
| Timeline | 3 (1-5) | 2 (0-3) | 3 (2-5) | 4 (3-6) | 5 (4-5) | <0.001 |
| Personal control | 5 (2-6) | 4 (1-6) | 4 (2-6) | 5 (4-6) | 5 (5-7) | 0.061 |
| Treatment control | 5 (4-8) | 5 (2-8) | 5 (3-9) | 5 (4-6) | 5 (4-7) | 0.99 |
| Identity | 4 (1-6) | 1 (0-3) | 4 (2-6) | 5 (4-7) | 6 (5-8) | <0.001 |
| Concern | 3 (1-6) | 1 (0-3) | 3 (1-6) | 5 (3-7) | 5 (3-8) | <0.001 |
| Comprehension | 4 (1-5) | 2 (0-5) | 3 (1-5) | 5 (4-6) | 3 (2-5) | 0.014 |
| Emotions | 4 (1-5) | 4 (0-5) | 4 (1-5) | 5 (3-7) | 5 (3-7) | 0.002 |
|  |  |  |  |  |  |  |
| **Month 6** |  |  |  |  |  |  |
| **N** | **242** | **54** | **111** | **55** | **22** |  |
| Consequences | 4 (1-7) | 1 (0-4) | 3 (1-6) | 5 (2-8) | 6 (5-9) | <0.001 |
| Timeline | 3 (1-5) | 1 (0-3) | 3 (1-5) | 4 (3-6) | 5 (5-6) | <0.001 |
| Personal control | 4 (1-6) | 2 (0-5) | 4 (1-5) | 5 (2-7) | 5 (5-7) | 0.001 |
| Treatment control | 5 (3-9) | 8 (2-10) | 5 (2-9) | 5 (3-6) | 5 (3-8) | 0.11 |
| Identity | 2 (1-6) | 0 (0-1) | 2 (1-5) | 5 (2-6) | 7 (5-8) | <0.001 |
| Concern | 2 (0-5) | 0 (0-1) | 2 (0-6) | 3 (2-6) | 5 (3-8) | <0.001 |
| Comprehension | 3 (1-5) | 1 (0-5) | 3 (2-5) | 4 (1-7) | 5 (3-7) | <0.001 |
| Emotions | 2 (1-5) | 1 (0-3) | 2 (0-5) | 4 (2-5) | 6 (3-7) | <0.001 |
|  |  |  |  |  |  |  |
| **Month 12** |  |  |  |  |  |  |
| **N** | **223** | **52** | **101** | **49** | **21** |  |
| Consequences | 2 (0-6) | 1 (0-2) | 2 (0-6) | 5 (2-8) | 7 (5-9) | <0.001 |
| Timeline | 2 (0-5) | 0 (0-2) | 2 (0-5) | 5 (2-7) | 5 (4-7) | <0.001 |
| Personal control | 4 (1-7) | 5 (1-10) | 3 (0-5) | 5 (2-7) | 5 (5-7) | 0.009 |
| Treatment control | 5 (2-9) | 5 (2-10) | 5 (2-9) | 5 (2-6) | 5 (5-7) | 0.45 |
| Identity | 2 (0-6) | 0 (0-1) | 1 (0-6) | 4 (1-6) | 5 (4-8) | <0.001 |
| Concern | 2 (0-5) | 0 (0-1) | 1 (0-5) | 4 (1-6) | 5 (3-8) | <0.001 |
| Comprehension | 4 (1-6) | 2 (0-5) | 4 (1-5) | 4 (2-6) | 5 (4-7) | 0.018 |
| Emotions | 1 (0-5) | 0 (0-2) | 1 (0-5) | 3 (1-6) | 6 (4-7) | <0.001 |

P-value denotes outcome of Kruskal-Wallis tests. Long COVID symptoms were defined as those developing within 1 month of overall illness onset, in order to exclude sporadic symptoms that were less likely to be attributed to the consequences of COVID-19. Trajectory group membership based on the maximum *a posteriori* probability of belonging to that group.

**Supplementary Table S8. Multivariable linear mixed-effect model of determinants of higher total illness perception questionnaire (B-IPQ) scores over time (month 1, 6 and 12 since illness onset)**

|  | **Coef.** | **95% CI** | | **p-value** |
| --- | --- | --- | --- | --- |
|  |  | **Lower limit** | **Upper limit** |  |
| **Time since illness onset, months** |  |  |  | 0.001 |
| 1 | Ref. |  |  |  |
| 6 | -5.64 | -9.07 | -2.22 |  |
| 12 | -5.82 | -9.22 | -2.43 |  |
|  |  |  |  |  |
| **Trajectory group** † |  |  |  | <0.001 |
| Trajectory 1 | Ref. |  |  |  |
| Trajectory 2 | 6.73 | 2.64 | 10.81 |  |
| Trajectory 3 | 17.67 | 12.70 | 22.64 |  |
| Trajectory 4 | 19.15 | 12.95 | 25.35 |  |
|  |  |  |  |  |
| **Age, years (per 10 year increase)** | 0.74 | -0.14 | 1.63 | 0.130 |
|  |  |  |  |  |
| **Sex** |  |  |  | 0.749 |
| Male | Ref. |  |  |  |
| Female | 0.63 | -2.06 | 3.32 |  |
|  |  |  |  |  |
| **Timing of infection** |  |  |  | <0.001 |
| First wave | Ref. |  |  |  |
| Subsequent waves | -7.40 | -10.27 | -4.54 |  |
|  |  |  |  |  |
| **Initial COVID-19 severity** |  |  |  | <0.001 |
| Mild | Ref. |  |  |  |
| Moderate | 6.26 | 3.27 | 9.26 |  |
| Severe/critical | 6.51 | 2.80 | 10.22 |  |

According to Likelihood Ratio Tests, BMI category did not lead to a significantly better fit and was therefore excluded.

Timing of SARS-CoV-2 infection defined as: first wave (up to 1 June 2020) and subsequent waves (on or after 1 June 2020). Clinical severity defined as: mild as having an RR <20/min and SpO2 on room air >94% at both D0 and D7; moderate disease as having a RR 20–30/minutes, SpO2 90–94% and/or receiving oxygen therapy at D0 or D7; severe disease as having a RR >30/minutes or SpO2 <90% at D0 or D7; critical disease as requiring ICU admission. Long COVID symptoms were defined as those developing within 1 month of overall illness onset, in order to exclude sporadic symptoms that were less likely to be attributed to the consequences of COVID-19.

† Trajectory group membership was based on the maximum *a posteriori* probability of belonging to that group over a two-year period since illness onset.

**Supplementary Table S9. Association between month 1 total B-IPQ score and group-based trajectory group, adjusted for age, sex and timing of SARS-CoV-2 infection**

|  |  | **95% CI** | |  |
| --- | --- | --- | --- | --- |
|  | **aOR** | **Lower limit** | **Upper limit** | **p-value** |
| B-IPQ, total score (per 10 point increase)† | 2.35 | 1.85 | 2.99 | <0.001 |
| Age, years (per 10 year increase) | 1.00 | 0.84 | 1.20 | 0.966 |
| Sex |  |  |  |  |
| Male | Ref. |  |  |  |
| Female | 3.14 | 1.79 | 5.53 | <0.001 |
| Timing of infection‡ |  |  |  |  |
| First wave | Ref. |  |  |  |
| Subsequent waves | 4.07 | 1.83 | 9.04 | 0.001 |

B-IPQ = Brief Illness Perception Questionnaire; CI= confidence interval. aOR= adjusted odds ratio.

According to Likelihood Ratio Tests, BMI category and initial COVID-19 severity did not lead to a significantly better fit and were therefore excluded.

†The adjusted odds ratio demonstrates the odds of belonging to a 1-unit higher trajectory group, per 10-point increase in B-IPQ.

‡Timing of SARS-CoV-2 infection defined as: first wave (up to 1 June 2020) and subsequent waves (on or after 1 June 2020).

The model met the proportional odds assumption.

**Supplementary Figure S1. Group-based trajectories of number of any symptoms reported at 2-24 months after illness onset, adjusted for age (years), sex, BMI category and timing of infection (first wave versus subsequent waves)**

Central shape denotes mean number of symptoms; dashed lines represent the 95% confidence interval. Percentages in legend show the proportion of study participants belonging to the trajectory.

**Supplementary Figure S2**. *A posteriori* probability of symptom trajectory membership, by trajectory group

## References

1. WHO. A clinical case definition of post COVID-19 condition by a Delphi consensus. World Health Organization (WHO) clinical case definition working group on post COVID-19 condition **2021**.

2. WHO. Clinical management of COVID-19, **2021** 27 May
